# Close approximations to the sound of a cochlear implant

**DOI:** 10.3389/fnhum.2024.1434786

**Published:** 2024-07-17

**Authors:** Michael F. Dorman, Sarah C. Natale, Joshua S. Stohl, Jenna Felder

**Affiliations:** ^1^College of Health Solutions, Speech and Hearing Science, Arizona State University, Tempe, AZ, United States; ^2^North American Research Laboratory, MED-EL, Durham, NC, United States

**Keywords:** cochlear implant, single-sided deafness, sound quality, CI signal processing, perception of voice

## Abstract

Cochlear implant (CI) systems differ in terms of electrode design and signal processing. It is likely that patients fit with different implant systems will experience different percepts when presented speech via their implant. The sound quality of speech can be evaluated by asking single-sided-deaf (SSD) listeners fit with a cochlear implant (CI) to modify clean signals presented to their typically hearing ear to match the sound quality of signals presented to their CI ear. In this paper, we describe very close matches to CI sound quality, i.e., similarity ratings of 9.5 to 10 on a 10-point scale, by ten patients fit with a 28 mm electrode array and MED EL signal processing. The modifications required to make close approximations to CI sound quality fell into two groups: One consisted of a restricted frequency bandwidth and spectral smearing while a second was characterized by a wide bandwidth and no spectral smearing. Both sets of modifications were different from those found for patients with shorter electrode arrays who chose upshifts in voice pitch and formant frequencies to match CI sound quality. The data from matching-based metrics of CI sound quality document that speech sound-quality differs for patients fit with different CIs and among patients fit with the same CI.

## Introduction

In a recent paper, Dorman et al. (2022) described the perceived sound quality of a cochlear implant for single-sided deaf (SSD) patients fit with a relatively short (18.5 mm) electrode array. These sound-quality judgements were obtained by first playing a clean signal to the implanted ear and then asking the patient to alter a clean signal to the typical hearing (TH) ear in order to match the sound quality of the signal presented to the implanted ear. Potential modifications to the clean signal included changes in the (i) voice pitch (F0), (ii) intonation contour, (iii) location of formant frequencies, (iv) signal bandwidth and (v) width of spectral peaks (‘smear’). Other potential modifications included adding (i) a metallic sound quality, (ii) a ‘flanged’ sound quality (a slight frequency and amplitude shift over time) and (iii) a noise-band or sine vocoder signal. Guided by an experimenter, the patient indicated which modifications were needed to make the signal directed to the TH ear sound like the signal directed to the implanted ear. Similarity was rated on a 10-point scale with 10 indicating a complete match to implant sound quality. Three patients gave very high match ratings, i.e., 9.7, 9.8 and 9.9, indicating that the matches were very close to the sound of the CI signal. These matches were characterized by upshifts in the formant frequencies and/or F0. Formant frequency increases ranged from 100 Hz to 320 Hz and F0 increases ranged from 10 to 80 Hz. The upshifts are consistent with electrical stimulation from a relatively short electrode array that does not extend to the apex, or low frequency region, of the cochlea (Landsberger et al., 2015).

In this paper, we describe close matches, i.e., 9.5 and above, to CI sound quality for 10 patients fit with a relatively long (28 mm) electrode array. Our aim was to determine the modifications necessary to create close approximations to CI sound quality and to compare those modifications to the modifications chosen by patients fit with a shorter electrode array as reported in Dorman et al. (2022).

## Materials and methods

### Participants

Eight female listeners and two male listeners were identified from a sample of 30 MED-EL SSD-CI patients on the basis of providing a similarity score of 9.5 or greater on the matching task between the sound of their CI and the sound of the signal created for their TH ear. As shown in Table 1, six were fitted with the FS4 processing strategy, two with the FS4-p strategy and two with the FSP strategy (see Riss et al., 2014 for a description of these strategies). The default lower- and upper-frequency limits of the filter bank were 70 and 8,500 Hz, respectively.

**Table 1:** Demographic data for patients.

Patient	Gender	Processing strategy	Speech recognition quiet score (%)	Age	Etiology	Duration of deafness (years)	Duration of CI use (years)	Number of active electrodes	PTA 0.5, 1, 2, 4 kHz
1	F	**FS4p**	AzB 100	17	Viral Infection	1.6	2.3	11	6
2	M	**FS4**	AzB 90	55	Labyrinthitis	1	2	11	21
3	F	**FS4**	CNC 40	60	SSN	1	0.75	12	NA
4	M	**FS4**	AzB 76	48	Unknown	5.9	0.2	12	19
5	F	**FSP**	95 (pediatric)	12	Unknown	4.1	2.7	11	1
6	F	**FS4**	AzB 44	42	Unknown	13	2.2	11	6
7	F	**FS4**	CNC 48	57	SSN	0.3	4	12	16
8	F	**FS4p**	CNC 72	64	SSN	1	7	10	8
9	F	**FSP**	CNC 40	66	SSN	1	2	10	20
10	F	**FS4**	CNC 48	53	Meniere’s	7	6	12	NA

The pure-tone average (PTA) is for the not-implanted ear. NA, not available.

The patients’ ages ranged from 12 to 66 with a mean age of 47 years. The duration of deafness before implant ranged from 0.3 years to 13 years. At the time of testing, the duration of CI use ranged from 0.2 years to 7.0 years. Etiologies included viral infection (1) Meniere’s disease (1) and Labyrinthitis (1). For three patients the etiology was unknown. Four patients experienced a sudden, sensorineural hearing loss of unknown origin. The patients received a 28 mm MED-EL electrode array. By surgeon report, all were fully inserted. That said, the lowest frequency stimulated would have varied across the participants due to differences in the overall length of the cochlea (e.g., Landsberger et al., 2015). Electrode position, derived from CT imaging, was not available for this group of patients.

The patient data were extracted from different studies in our two laboratories on CI sound quality. For that reason, not all patients were tested with the same test of speech understanding. Six patients had CNC scores ranging from 40–72% correct and four had scores from the AzBio sentence test ranging from 44—100% correct. For context, Gifford et al. (2018) found, for a sample of approximately 500 CI patients, a mean CNC score of 52% correct and a mean AzBio score of 63% correct.

### Test signals

Two, male-voice sentences from the CUNY sentence corpus were used for testing: “Do you like camping?” and “The sun is finally shining”. The sentences were first synthesized using the STRAIGHT algorithm (Kawahara et al., 2001) so that other manipulations could be implemented efficiently. Details of the synthesis can be found in Dorman et al. (2022).

Custom-built software produced changes in the acoustic characteristics of each sentence in order to create candidate sentences for the NH ear. Sound changing operations could be implemented, via an on/off toggle, singly or in any combination. At output, signal modifications were implemented in the order described below.

The mean fundamental frequency of the voice (F0) could be increased or decreased and the F0 contour (i.e., the intonation contour) could be flattened in steps from 100 to 0% of the normal extent.

Formant frequencies could be shifted over the range − 50 Hz to +300 Hz. In our implementation, the difference in frequency between formants was maintained and the whole spectrum was shifted up or down in frequency linearly.

Spectral peaks could be broadened and spectral peak-to-valley differences reduced in a simulation of the effects of poor frequency selectivity (algorithm modeled after Baer and Moore, 1993).

Noise and sine vocoders could be implemented with 4–12 channels (see Dorman et al., 2017). Both types of vocoder outputs could be combined with a non-vocoded signal.

A slight frequency and amplitude shift over time was implemented by creating a signal that was 0.01% longer than the original signal and then combining the two signals. Perceptually the combined signal sounded slightly comb filtered or “flanged”.

Signals could be given a metallic sound quality by altering resonances and ring times. A filter was constructed using a bank of sharp, inharmonically-related resonances in combination with a bandpass filter (0.442 to 4.248 kHz).

Signals could be low-, high- and band-pass filtered using 6th order Butterworth filters with variable corner frequencies.

Additional details on the operations described above can be found in Dorman et al. (2022).

### Procedure

The procedure used for patient testing is shown in videos in Dorman et al. (2019a,b, 2020) and is described in detail in Dorman et al. (2022). Briefly, signals were delivered to the CI via a direct connect cable and signals were delivered to the TH ear via an insert receiver (ER3-A) or a single-ear headphone (Beyerdynamic DT 252). A clean signal was delivered to the CI first and then to the TH ear. The experimenter asked the patient how to modify the signal to the TH ear to match the sound quality of the signal presented to the CI ear.

This process, asking the patient what needed to be changed and then altering the signal, continued until the patient said that the match was ‘very close’ and/or the parameter set had been exhausted. At this point, the patient was asked to rate the similarity of the signal presented to the TH ear relative to that of the CI on a 10-point scale with 10 being a complete match. Testing took approximately 30 min to complete.

All procedures were approved by WCG^™^ IRB.

## Results

**Figure 1:**
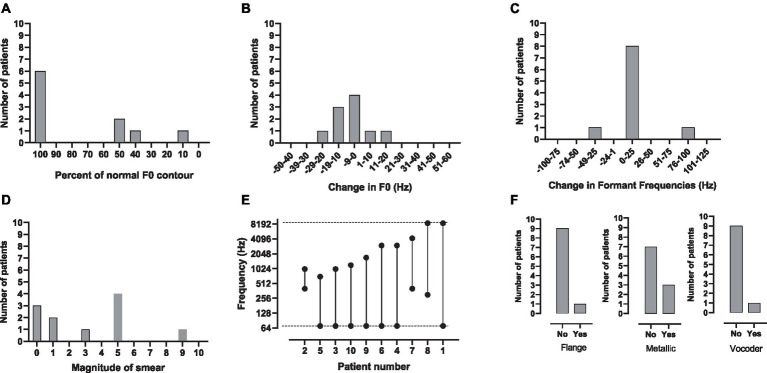
**(A–F)** The number of patients who chose a given parameter setting for **(A)** percent of normal F0 contour, **(B)** change in F0, **(C)** change in formant frequencies, **(D)** spectral smear, **(E)** bandwidth and **(F)** flange, metallic and vocoder. In **E**, the dotted lines indicate the default lower- and upper-frequency limits of the signal-processor filter-bank, 70 and 8,500 Hz, respectively.

The parameters used to create a match to CI sound quality are shown in Table 2 and Figures 1A–F. Figure 1F shows the small number of patients who needed the (i) flange, (ii) metallic and (iii) vocoder operations to match CI sound quality. One patient requested flange, three requested metallic and one requested a sine vocoder. Another infrequently used modification was a change in formant frequencies (Figure 1C). Only two patients requested this – one wanted a downward shift (−25 Hz) and one an upward shift (100 Hz). Flattening of the F0 contour (Figure 1A) – was requested by five patients. The flattening ranged from 90% of normal to 10% of normal.

**Table 2:** Modifications to a clean signal directed to the TH ear used to match the sound quality of signals directed to the implanted ear.

Patient	F0 contour	Change in F0 (Hz)	Change in formant freq. (Hz)	Smear	Flange	Metallic	Filtering	Vocoder	Match rating
1	No change	−10	No change	5	No	No	none	No	10
2	50%	−10	No change	5	No	No	BP 0.4–1.0 kHz	No	10
3	40%	No change	No change	5	No	Yes	LPF 1.0 kHz	No	9.9
4	10%	No change	No change	none	No	No	LP 3.0 kHz	Sine VC -6 dB	9.5
5	No change	5	No change	none	No	No	LP 0.7 kHz	No	9.5
6	50%	20	No change	none	Yes	No	LP 3.0 kHz	No	9.5
7	No change	−15	No change	3	No	Yes	BP 0.4–4.2 kHz	No	9.5
8	No change	No change	100	1	No	No	HPF 0.3 kHz	No	9.5
9	No change	−29	−25	9	No	No	LPF 1.7 kHz	No	9.5
10	90%	No change	No change	4.5	No	Yes	LPF 1.2 kHz	No	9.5

The most commonly used modifications were a change in F0, smear and filtering. As shown in Figure 1B for F0, four of the patients asked for no modification, four wanted a downward shift (−10, −10, −15, −29 Hz) and two an upwards shift (5, 20 Hz). As shown in Figure 1D, seven patients wanted some amount of smear; two minimal, four moderate and one maximal. The most commonly requested modification was filtering. As shown in Figure 1E, nine patients wanted some type of filtering – most commonly low-pass. The most-narrow bandwidth was 0.6 kHz, the widest 8.53 kHz.

Patients who requested a relatively narrow bandwidth (BW), i.e., 0.6 kHz to 1.63 kHz, to match the sound of their CI also requested at least a moderate degree of smear. Patients 2 (BW = 0.6 kHz; Smear = 5), 3 (BW = 0.93 kHz; Smear = 5), 9 (BW = 1.63 kHz; Smear 8.9) and 10 (BW = 1.13 kHz; Smear 4.5) fit this pattern. On the other hand, patients who requested a relatively wideband signal (>/= 2.93 kHz) to match the sound of their CI requested minimal or no smear. Patients 4 (BW = 2.93 kHz, Smear = 0), 6 (BW = 2.93 kHz; Smear = 0), 7 (BW = 3.8 kHz; Smear 3), and 8 (BW = 8.2 kHz; Smear = 1) fit this pattern. These patients chose a variety of other manipulations to make the signals sound like their CI. Small changes in F0 were chosen by half of the patients. Other manipulations were chosen by only one or two patients. The audio files for the matches to CI sound quality for all patients can be found in Supplementary material.

## Discussion

As indicated in the introduction, the aim of this project was to determine the modifications necessary to create very close approximations to CI sound quality for patients fit with a relatively long electrode array and to compare those modifications to the modifications chosen by patients fit with a shorter electrode array as reported in Dorman et al. (2022). Because three of the patients with short arrays in Dorman et al. (2022) gave very high match ratings of 9.7–9.9, the patients in the present study were also chosen to have high ratings (9.5–10).

In Dorman et al. (2022), the patients with the highest match scores chose an upward shift in formant frequencies ranging from 100 Hz to 320 Hz combined with upward shifts in F0 ranging from 10 Hz to 80 Hz to match CI sound quality. These shifts yielded a ‘high pitched’ voice quality consistent with electrode arrays that did not extend to the low frequency portion of the cochlea. In contrast, in the present study with patients fit with a longer electrode array, only one patient wanted an upshift in formant frequencies and two wanted upshifts in F0. Changes in F0 were more likely to be negative than positive. As a consequence, the voice quality in Dorman et al. (2022) is not representative of the voice quality for patients in the present study.

In the present study, two response patterns were identified. Five of the patients asked for a narrow bandwidth — 0.6 to 1.6 kHz — and four of these asked for at least moderate smear. An additional patient asked for a narrow bandwidth (0.6 kHz) but no smear. The stimulus dimensions picked by these patients suggest a muffled sound quality. In contrast, four of the patients picked either no filtering or at least a 2.9 kHz bandwidth and no or minimal smear to create a match. An additional patient asked for an 8 kHz bandwidth and moderate smear. The minimal or absent band limiting and smear suggests a percept that was not as muffled as that experienced by the other patients.

It would be reasonable to suspect that patients who asked for a narrow bandwidth would have a compressed range of electrode pitches. To explore this, Patient 2, who matched CI sound quality to a signal that was high-pass filtered at 0.4 kHz and low-pass filtered at 1.0 kHz, was asked to match the pitch of sine signals presented to electrodes E1, E4, E7 and E10 (E1 was the most apical electrode). Stimulation at E1 was matched to 110 Hz while stimulation at E10 was matched to 3,575 Hz. Thus, the patient’s match to a very narrow bandwidth in speech sound-quality testing was not the result of a compressed range of electrode pitch. Given this outcome, it is possible that the perceptual effect of a narrow bandwidth in the TH ear mimics the perceptual effect of other abnormalities in signal processing that coincide with electrical stimulation of the implanted ear.

Another patient, in the 30-patient sample from which we extracted the data for this paper, gave a match rating of 9.5 but was not included in the data described above because his electrode array was not fully inserted. We describe his results here because his speech quality matches make us question the inference from our previous data that shorter electrode arrays always lead to upshifted percepts. High resolution CT scans showed only nine electrodes in the cochlea and seven in the range of speech frequencies. OTOPLAN software (see review by Gatto et al., 2023) indicated that Electrode 1 (E1) was at the 957 Hz spiral ganglion (SG) frequency, E4 at 2393 Hz and E7 at 7173 Hz. As a consequence, it was reasonable to suspect that both electrode pitch and speech quality matches would be upshifted. This was not the case. Pitch matching at E1 (957 Hz SG frequency) resulted in a match at 109 Hz, at E4 (2,393 Hz SG frequency) at 628 Hz and at E7 (7,173 SG frequency) at 4851 Hz. The 9.5 matching score was obtained with a signal created with a high-pass filter at 160 Hz, i.e., much lower than the SG frequency of 957 Hz. The patient indicated that, even at the time of device activation, speech signals did not sound upshifted in frequency or like Mickey Mouse^™^. If this was, in fact, the case, then there are patients who, from the time of device activation, respond primarily to temporal coding of frequency, i.e., the stimulation frequency, rather than place (or SG) coding of frequency. One perceptual consequence of this would be minimal, or absent, Mickey Mouse^™^ voice-quality.

Another account for pitch matches lower than the SG frequencies is the phenomenon of adaptation, i.e., the change, most generally a lowering, in perceived electrode pitch over time in which the pitch comes to approximate the center frequency of the energy in the filter in front of the electrode (Reiss et al., 2007; McDermott et al., 2009; Tan et al., 2017). However, this effect generally takes time and our patient indicated that the signal was not heard as upshifted even near the time of device activation.

Overall, the several papers in this series (Dorman et al., 2019a,b, 2020, 2022) document different speech sound-quality percepts for patients fit with longer and shorter electrode arrays as well as different percepts for patients fit with the same electrode array and signal processing algorithm. The difference in CI sound-quality between groups of patients with longer and shorter arrays can be related to the differences in extent of low-frequency spiral ganglion neurons stimulated by the electrode arrays. The factors contributing to differences in speech sound quality for patients fit with the same electrode array and signal processing remain to be discovered. The outcome that scores of 10, i.e., a perfect match, were very rare suggests that (i) the number and nature of the dimensions we employ are not sufficient to completely capture the sound quality of a CI, (ii) there are effects of electric stimulation which cannot be approximated by an acoustic signal to a normal-hearing ear or (iii) both (i) and (ii). If the latter is the case, then scores near 9.5 but below 10 will be the best we can do.

## Data availability statement

The original contributions presented in the study are included in the article/Supplementary material, further inquiries can be directed to the corresponding author.

## Ethics statement

The studies involving humans were approved by WCG^™^ (previously the Western Institutional Review Board) protocol #20100066. The studies were conducted in accordance with the local legislation and institutional requirements. The participants provided their written informed consent to participate in this study.

## Author contributions

MD: Writing – original draft, Writing – review & editing, Conceptualization, Formal analysis, Methodology. SN: Data curation, Methodology, Software, Supervision, Writing – review & editing. JS: Conceptualization, Data curation, Formal analysis, Funding acquisition, Investigation, Methodology, Software, Writing – review & editing. JF: Data curation, Formal analysis, Investigation, Project administration, Supervision, Writing – review & editing.
